# The brain-enriched microRNA miR-124 in plasma predicts neurological outcome after cardiac arrest

**DOI:** 10.1186/cc13753

**Published:** 2014-03-03

**Authors:** Patrik Gilje, Olof Gidlöf, Malin Rundgren, Tobias Cronberg, Mariam Al-Mashat, Björn Olde, Hans Friberg, David Erlinge

**Affiliations:** 1 Department of Cardiology, Lund University, Skane University Hospital, 221 85 Lund, Sweden; 2 Department of Intensive and Perioperative Care, Lund University, Skane University Hospital, 221 85 Lund, Sweden; 3 Department of Clinical Sciences, Division of Neurology, Lund University, Skane University Hospital, 221 85 Lund, Sweden; 4 Department of Clinical Physiology, Skåne University Hospital, 221 85 Lund, Sweden

## Abstract

**Introduction:**

Early prognostication after successful cardiopulmonary resuscitation is difficult, and there is a need for novel methods to estimate the extent of brain injury and predict outcome. In this study, we evaluated the impact of the cardiac arrest syndrome on the plasma levels of selected tissue-specific microRNAs (miRNAs) and assessed their ability to prognosticate death and neurological disability.

**Methods:**

We included 65 patients treated with hypothermia after cardiac arrest in the study. Blood samples were obtained at 24 hours and at 48 hours. For miRNA-screening purposes, custom quantitative polymerase chain reaction (qPCR) panels were first used. Thereafter individual miRNAs were assessed at 48 hours with qPCR. miRNAs that successfully predicted prognosis at 48 hours were further analysed at 24 hours. Outcomes were measured according to the Cerebral Performance Category (CPC) score at 6 months after cardiac arrest and stratified into good (CPC score 1 or 2) or poor (CPC scores 3 to 5).

**Results:**

At 48 hours, miR-146a, miR-122, miR-208b, miR-21, miR-9 and miR-128 did not differ between the good and poor neurological outcome groups. In contrast, miR-124 was significantly elevated in patients with poor outcomes compared with those with favourable outcomes (*P* < 0.0001) at 24 hours and 48 hours after cardiac arrest. Analysis of receiver operating characteristic curves at 24 and 48 hours after cardiac arrest showed areas under the curve of 0.87 (95% confidence interval (CI) = 0.79 to 0.96) and 0.89 (95% CI = 0.80 to 0.97), respectively.

**Conclusions:**

The brain-enriched miRNA miR-124 is a promising novel biomarker for prediction of neurological prognosis following cardiac arrest.

## Introduction

Sudden cardiac arrest is a cause of substantial mortality and morbidity that affects more than one million individuals worldwide every year [1]. Early prognostication of neurological outcome after successful cardiopulmonary resuscitation using clinical examination alone is difficult and has become more complicated with the introduction of therapeutic hypothermia as a treatment strategy for comatose cardiac arrest patients. Hypothermia affects the metabolism of sedative drugs, and lingering sedation has been found to be an important reason for the decreased reliability of a clinical neurological examination to predict poor outcome after cardiac arrest, as reported by several authors [2,3]. Hence, there is a need for novel methods to estimate the extent of brain injury and predict outcome. Several circulating biomarkers, such as neuron-specific enolase (NSE), S100B and neurofilament heavy chain levels, have been evaluated with regard to prognosis. These assessments have revealed varying degrees of success, and the search for an ideal biomarker continues [4-6]. The modern approach for prognostication after cardiac arrest combines a neurological examination comprising neurophysiology, biochemical tests and imaging several days after the incident [7].

MicroRNAs (miRNAs) are short (18 to 23 nucleotides), noncoding RNAs that have a central role in regulating translation and degradation of mRNAs in the cell [8]. By complementary base-pairing with the 3′ untranslated region of a specific mRNA, miRNAs suppress gene expression and affect a wide range of physiologic and pathologic processes. Some miRNAs have a high degree of tissue specificity, and more than 2,000 human microRNAs have been characterized to date [9,10]. In animal models, cerebral ischemia and reperfusion alter the tissue expression of several miRNAs that are known to be associated with modulation of apoptosis [11,12], promotion of cell death [13] and cell-cycle regulation [14], thereby potentially influencing outcomes.

In recent years, miRNAs have been detected in plasma, where they are associated with proteins and extracellular vesicles and have been found to be remarkably stable, remaining in the circulation for days [12]. Furthermore, many miRNAs have a high degree of organ specificity, thus making them suitable markers of organ-selective tissue injury. After organ-related injuries such as myocardial infarction, hepatitis and stroke, levels of tissue-specific miRNAs increase in the circulation, thus indicating a role for miRNAs as clinically useful biomarkers [15-17]. In animal models, the highly brain-enriched miRNA miR-124 [9] has been put forward as a marker of ischemic brain damage [17,18], but research on human samples after brain injury is relatively scarce. Investigators in one study found a difference in plasma miRNA profiles between stroke patients and controls as well as between patients with different stroke aetiologies [19], but the potential role of miRNAs as prognostic markers for stroke has yet to be clarified. In the field of cardiac arrest, however, researchers in a recent study of 28 patients showed that miR-122 and miR-21 in plasma correlated to long-term prognosis 48 hours after cardiac arrest [20].

Our aim in this study was to evaluate the impact of the cardiac arrest syndrome on the plasma levels of selected tissue-specific miRNAs and to assess their ability to accurately prognosticate death and neurological disability. Researchers in previous studies have focused on tissue samples taken 48 hours after cardiac arrest, so we initially assessed this time point. Because of the promising results of miR-124, this miRNA was further analysed at 24 hours and compared with miR-124 in age- and sex-matched controls.

## Materials and methods

### Ethical approval

The study was approved by the Regional Ethical Review Board at Lund University (411/2004 and 223/2008). Informed consent was sought from the patient’s next of kin or retrospectively from the patient.

### Study protocol

A more detailed version of the materials and methods can be found in the Additional file 1. This study was performed between June 2003 and March 2008 and included unconscious patients resuscitated after cardiac arrest, regardless of location or initial cardiac rhythm. Unwitnessed asystole was an exclusion criterion. Computed tomography of the brain was performed liberally at admission to exclude patients with intracranial bleeding. Serial samples were collected from consecutive patients treated with hypothermia within 6 hours after admission and at 24 ± 4 hours and 48 ± 4 hours after admission. The treatment protocol of this study has been described in detail elsewhere [4]. Briefly, all patients were treated with hypothermia for 24 hours at 33 ± 1°C, followed by rewarming at 0.5°C/h. In patients who remained unconscious, intensive care was continued for at least 72 hours after normothermia [21].

All surviving patients were followed up at 6 months after their cardiac arrest and categorized according to the five-point Cerebral Performance Category (CPC) scale: CPC score 1: good cerebral performance, CPC score 2: moderate cerebral disability, CPC score 3: severe cerebral disability, CPC score 4: coma and CPC score 5: death [22]. A CPC score of 1 or 2 at 6 months after cardiac arrest was considered a good outcome.

Ten patients were randomly selected, and the baseline levels of miR-124 were compared to those levels in age- and sex-matched controls (5 women and 5 men in each group, median ages 72 years in the control group and 73 years in the patient group; *P* = ns). The control group consisted of patients who underwent a myocardial perfusion scan because of suspicion of angina pectoris, and the samples were obtained before the scan. Because outcome was not correlated to miR-124 at baseline, we did not stratify the patients according to CPC group. Seven of the patients belonged to the good outcome group (CPC score 1 or 2) and three patients to the poor outcome group (CPC scores 3 to 5).

### RNA isolation and cDNA synthesis

The RNA was extracted according to the manufacturer’s instructions (QIAGEN, Hilden, Germany). RNA was then isolated with the miRNeasy Mini Kit (QIAGEN). For normalization purposes, a UniSp RNA Spike-in mix/sample (Exiqon A/S, Vedbæk, Denmark) was added before preparation of the samples. cDNA was synthesized with the miRCURY LNA Universal RT cDNA synthesis kit (Exiqon) using a fixed volume of RNA preparation in each reaction according to the manufacturer’s instructions. When preparing RNA from plasma, the yields are insufficient for proper quantitation with, for example, a NanoDrop spectrophotometer (Thermo Fisher Scientific, Wilmington, DE, USA) [23]. Therefore, equal volumes of RNA preparation, rather than equal RNA amounts, were used as input in the cDNA synthesis.

### Quantitative RT-PCR

Custom quantitative RT-PCR (qRT-PCR) panels, including 20 miRNAs, were used for screening purposes according to the manufacturer’s instructions (Exiqon) on a StepOnePlus Real-Time PCR System (Applied Biosystems, Foster City, CA, USA). Raw cycle threshold (Ct) values were normalized against the mean of the spike-in controls Sp2 and Sp4 to adjust for differences in RNA extraction and reverse transcription efficiencies, and the results are expressed relative to the mean of the baseline samples using the formula 2^-ΔΔCt^.

qRT-PCR was run using primer sets according to the protocol of the manufacturer (Exiqon) on the StepOnePlus Real-Time PCR System. Whenever possible, the results are expressed relative to baseline and the spike-in Sp2 using the 2^-ΔΔCt^ method. When we analysed the difference in miRNA levels at admission, however, we expressed the results relative to the spike-in Sp2 using the formula 2^-ΔCt^.

Initially, a miRNA-screening trial was performed on samples obtained at 48 hours after cardiac arrest from eight randomly selected patients from each of the two outcome groups. After we reviewed the literature, we identified 20 plasma miRNAs that we hypothesized would be of interest in studying the cardiac arrest syndrome, which we analysed on the custom panels described above. One plate containing tissue from two patients (one in each CPC category) was excluded because of a technical failure, and another patient in the CPC 1 or 2 group was excluded because of inhibition of the PCR as detected by the spike-in controls, leaving us with 13 patients for further analysis. After Bonferroni corrections, no significant differences were observed between the groups for any of the 20 miRNAs (see Additional file 2: Figure S1). On the basis of the visual inspection of the distributions and the results reported by Stammet *et al*. [20], however, we decided to assess the prognostic properties of the brain-enriched miR-124, miR-9 and miR-128; the inflammation-associated miR-146a; the liver-specific miR-122; the cardiac tissue–specific miR-208b; and the apoptosis inhibitor/angiogenesis-associated miR-21 on the entire cohort. The associated plasma proteins C-reactive protein (CRP), alanine aminotransferase (ALT) and troponin T (TnT) were measured. NSE values were gathered from a previous study of the same patient cohort [4].

Calculations and statistics were performed using GraphPad Prism 5 software (GraphPad Software La Jolla, CA, USA) and SPSS version 21 software (IBM SPSS, Inc, Chicago, IL, USA). Continuous data are presented as medians and ranges. Categorical variables are given as percentages. The patients were dichotomised into good and poor outcome groups according to CPC scores, using CPC score 1 or 2 as good outcomes and CPC scores 3 to 5 as poor. The distribution of miRNA levels in the two groups was assessed using the D’Agostino-Pearson omnibus normality test. As the data could not be described by a normal distribution, the two-tailed Mann–Whitney *U* test was used to detect differences in miRNA levels with regard to outcomes. Categorical variables were analysed using Fisher’s exact test. The ability of miR-124 to discriminate between good vs poor prognosis at 24 h and 48 h was assessed with receiver operating characteristic (ROC) curve analysis. Correlations were performed using Spearman’s correlation test. Bonferroni corrections were applied to the *P*-values.

## Results

The initial cohort consisted of 92 patients. We excluded 25 patients because of missing samples, and 2 patients were excluded because of unknown outcomes and/or missing patient data. In the end, 35 patients with favourable outcomes (CPC score 1 or 2) and 30 with poor outcomes (CPC scores 3 to 5) with tissue samples taken at baseline and 24 hours and 48 hours after cardiac arrest were entered into the study. The patients’ characteristics are summarized in Table 1. There were no differences between the outcome groups with regard to age, sex, initial cardiac rhythm or bystander cardiopulmonary resuscitation. Patients with poor outcomes had a longer time to return of spontaneous circulation and a longer time to initiation of hypothermia compared with those who had good outcomes.

**Table 1:** Patient characteristics in the CPC1-2 and CPC 3-5 cohorts^a^

**Characteristics**	**CPC 1-2 (*n*** **= 35)**	**CPC 3-5 (*n*** **= 30)**	***P*-value**
Age, (years)	65 (15 to 82)	72 (14 to 87)	ns
Female sex	9 (26%)	14 (47%)	ns
Initial rhythm			
VT/VF	28 (80%)	19 (63%)	ns
Asystole	3 (9%)	7 (23%)	ns
PEA	4 (11%)	4 (13%)	ns
Witnessed CA	29 (83%)	28 (93%)	ns
Bystander CPR	17 (49%)	11 (37%)	ns
Out-of-hospital CA	30 (86%)	22 (73%)	ns
Time from CA to ROSC (minutes)	16 (2 to 47)	25 (7 to 110)	<0.01
Time from CA to initiation of hypothermia (minutes)	60 (0 to 330)	70 (45 to 210)	<0.05
Time from CA to target temperature (minutes)	225 (25 to 565)	193 (100 to 430)	ns
Primary cardiac cause	28 (80%)	18 (60%)	ns

^a^CA, Cardiac arrest; CPC, Cerebral Performance Category; CPR, Cardiopulmonary resuscitation; ns, Not significant; PEA, Pulseless electrical activity; ROSC, Return of spontaneous circulation; VF, Ventricular fibrillation; VT, Ventricular tachycardia. Values are presented as median (range) or counts and percentages.

**Figure 1:**
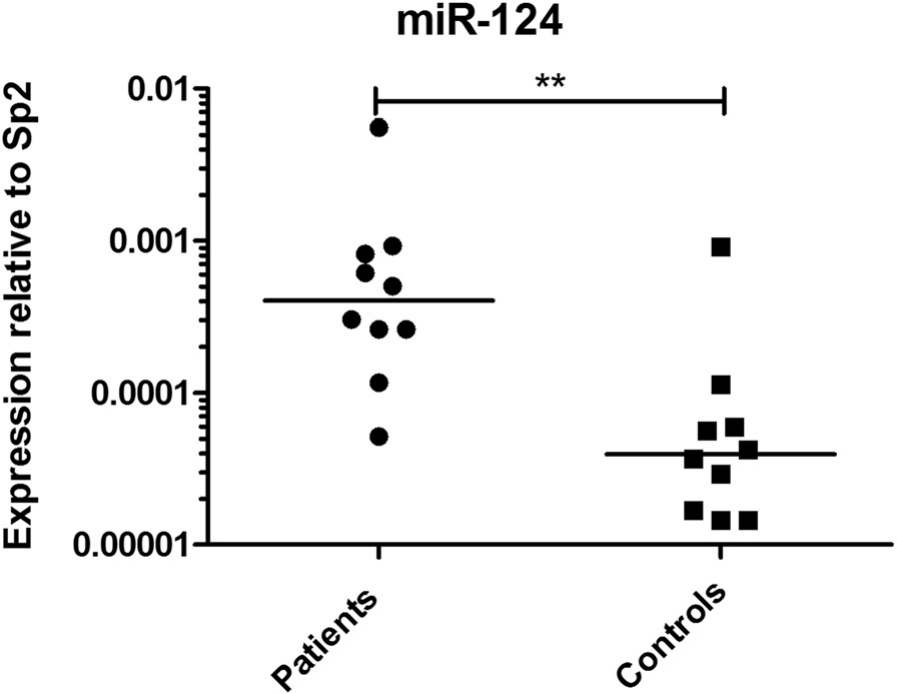
**Plasma microRNA-124 was already elevated tenfold at the time of admission in patients compared with controls.** The horizontal lines represent the medians. miR-124, MicroRNA-124.

**Figure 2:**
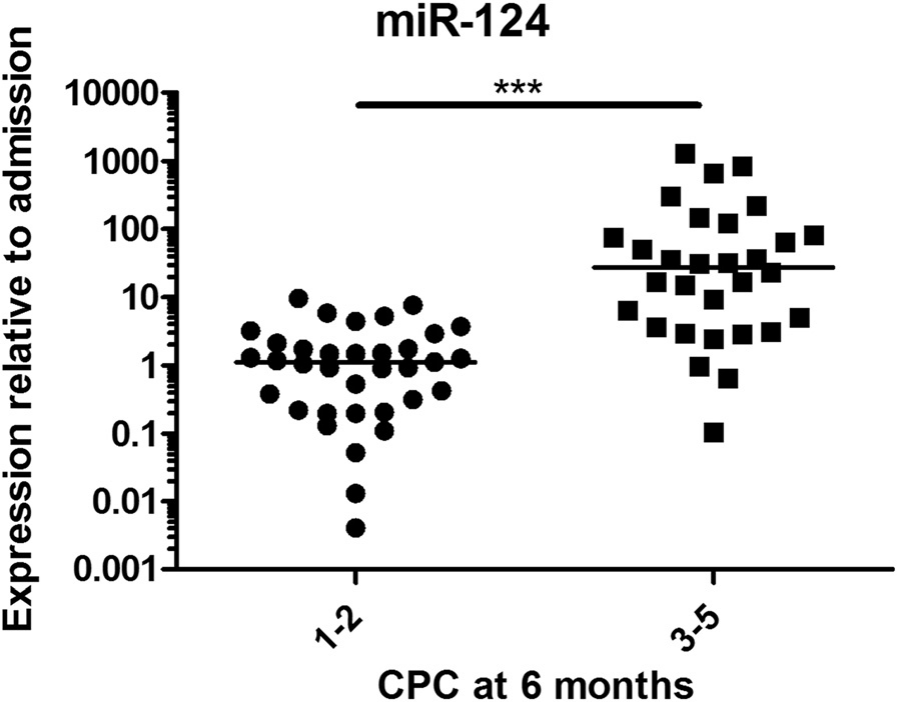
**A poor outcome was associated with a significant elevation of microRNA-124 at 48 hours after cardiac arrest.** The horizontal lines represent the medians. CPC, Cerebral Performance Category scale score; mIR-124, MicroRNA-124. ***P < 0.0001.

**Figure 3:**
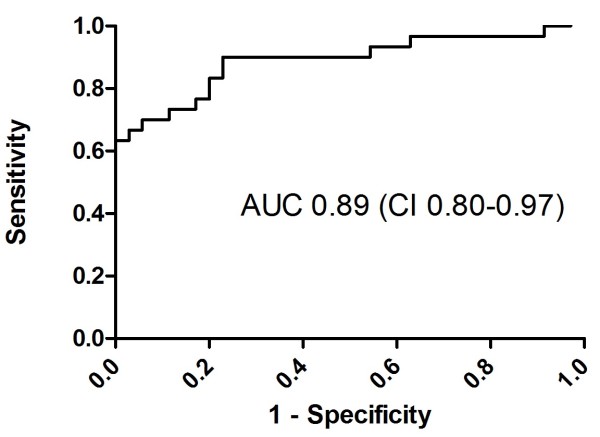
**Receiver operating characteristic area under the curve of microRNA-124 at 48 hours after cardiac arrest.** AUC, Area under the curve; CI, 95% confidence interval.

At admission within 6 hours after cardiac arrest, there were no significant differences in miRNA levels between the outcome groups (see Additional file 3: Figure S2). miR-124 levels were already elevated tenfold at admission in patients compared with age- and sex-matched controls (*P* < 0.01) (Figure 1). At 48 hours, miR-146a, miR-122, miR-208b, miR-21, miR-9 and miR-128 levels did not differ between the good and poor neurological outcome groups. In contrast, miR-124 was elevated 24-fold in patients with poor outcomes compared with those who had favourable outcomes (*P* < 0.0001) (Figure 2). Analysis of ROCs at 48 hours showed an AUC of 0.89 (95% CI = 0.80 to 0.97) (Figure 3). A cutoff of 12 yielded 100% specificity and 63% sensitivity at 48 hours for a poor neurological outcome.

**Figure 4:**
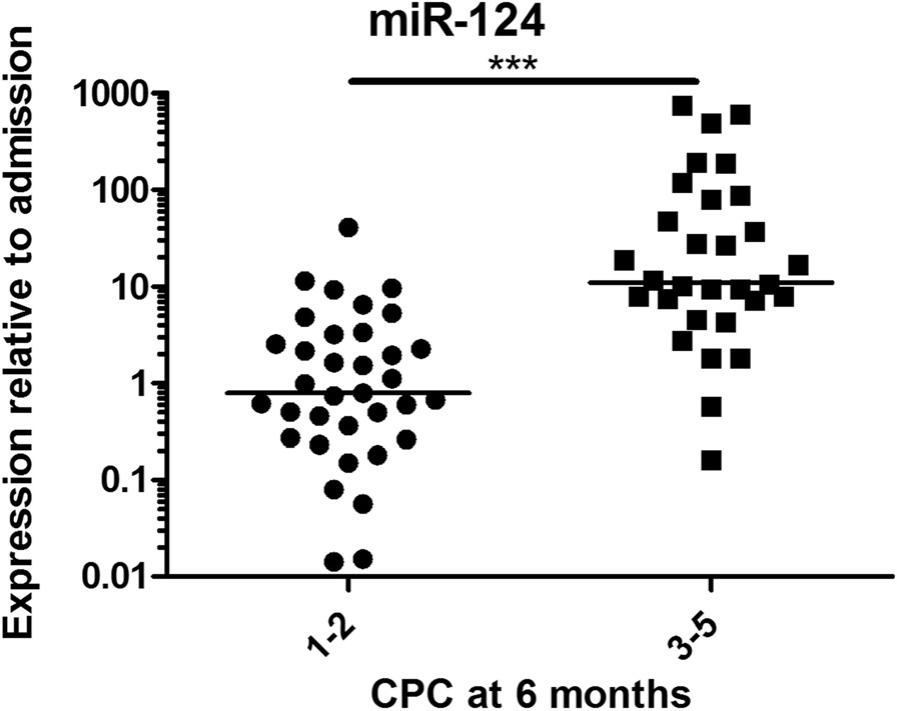
**Plasma microRNA-124 was significantly elevated in patients with poor outcomes at 24 hours after cardiac arrest.** The horizontal lines represent the medians. CPC, Cerebral Performance Category scale score; mIR-124, MicroRNA-124. ***P < 0.0001.

**Figure 5:**
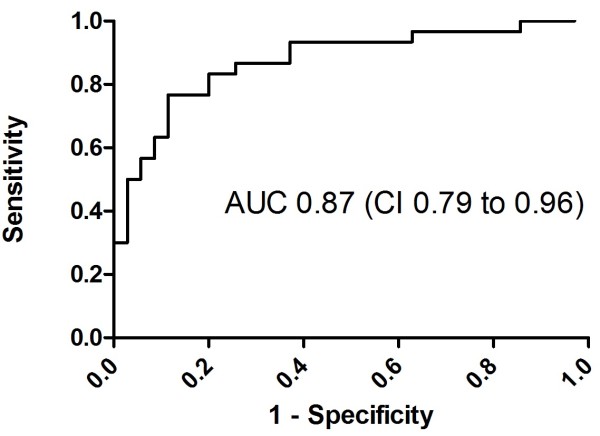
**Receiver operating characteristic area under the curve of microRNA-124 at 24 hours after cardiac arrest.** AUC, Area under the curve; CI, 95% confidence interval.

At 24 hours after cardiac arrest, the levels of miR-124 were 14-fold higher in the poor outcome group than in the good outcome group (*P* < 0.0001) (Figure 4). Furthermore, calculation of ROCs for a poor neurological prognosis resulted in an AUC of 0.87 (95% CI = 0.79 to 0.96) (Figure 5). A cutoff of 44 yielded 100% specificity and 30% sensitivity at 24 hours for a poor neurological outcome. However, a cutoff of 12 increased sensitivity to 53% while maintaining a specificity of 97% for a poor neurological outcome.

Analysis of ROC curves for NSE revealed AUCs of 0.90 (95% CI = 0.79 to 1.00) at 48 hours and 0.80 (95% CI = 0.67 to 0.93) at 24 hours, which are similar to the results for miR-124. Combining miR-124 and NSE did not improve diagnostic accuracy (AUC = 0.90 (95% CI = 0.80 to 1.00) at 24 hours and AUC = 0.93 (95% CI = 0.84 to 1.00) at 48 hours).

Levels of miR-124 correlated only moderately to NSE (*r* = 0.51 at 24 hours and *r* = 0.49 at 48 hours). No correlation was found at 48 hours between the immunologically active miR-146a and CRP. A poor correlation was found between the cardiac miR-208b and TnT (*r* = 0.44 at 48 hours). Similarly, miR-122 at admission was only weakly related to ALT at 24 hours (*r* = 0.49).

## Discussion

The results of our present study indicate that the brain-enriched miRNA miR-124 is a promising candidate marker to use in predicting outcomes after cardiac arrest. A cutoff of 12 at 24 hours yielded 97% specificity and 53% sensitivity for prognostication of CPC scores of 3 to 5, a result which may support its clinical efficacy. Moreover, miR-124 demonstrated good predictive properties at 48 hours, at which point a cutoff of 12 yielded 100% specificity and 63% sensitivity for a poor neurological outcome. As a biomarker of brain damage, miR-124 has previously been characterized in plasma after induction of stroke in rodents, but our present study, to the best of our knowledge, is the first in which miR-124 has been associated with brain injury in humans. miR-124 was comparable to NSE with regard to prognostication, and combining the two biomarkers did not improve diagnostic accuracy.

Our findings are of importance because early prognostication after cardiac arrest is difficult, mainly due to the liberal use of sedatives, analgesics and muscle relaxants. The previously accepted time point for prognostication—72 hours after cardiac arrest—is no longer valid, because, owing to the lingering effects of sedation, the reliability of the neurological examination cannot be assured [2,3]. Therefore, additional methods by which to assess the extent of brain injury are needed. Ideally, these methods should be independent of each other, inexpensive, easily interpreted and without significant sources of error. NSE is the only biomarker that has been recommended for prediction of outcomes after cardiac arrest [24], but its use has been limited by a lack of standardisation as well as diverging cutoff levels for prediction of poor prognosis in patients who receive hypothermia treatment and those who do not [6]. Another major drawback of NSE is that haemolysis, which may occur among cardiac arrest survivors, yields falsely elevated NSE levels [25].

We could not confirm the findings from the only previously published study in which the investigators studied miRNA levels in plasma after cardiac arrest [20]. In that prior study, miR-21 and miR-122 were put forward as prognostic markers in a cohort of 28 patients 48 hours after cardiac arrest. The study was small, however, and raised some issues. First, the levels of miR-124 were not investigated, despite being the most studied plasma miRNA in the setting of cerebral ischemia in animal models [17,18]. Second, microarrays were performed, clustering 115 miRNAs, but most data derived from this screening were not presented. Third, on the basis of an *in vitro* experiment using retinoic acid–differentiated neuroblastoma cells, the authors speculated that miR-122 might originate in the brain. However, it is not correct to equate miR-122 expression generated by artificial differentiation with a true neural phenotype. Furthermore, miR-122 is generally regarded as a liver-specific miRNA, and to date no authors have reported any significant levels of miR-122 in the human brain [26]. In our present study, which included more patients, we are unable to confirm that miR-122 has prognostic properties after cardiac arrest. Instead, we found a decrease in the plasma levels of miR-122 in both outcome groups, which is in concordance with data in published reports on patients with myocardial infarction as well as experiments on rats exposed to transient brain ischemia [23,27]. Moreover, we were not able to repeat the finding of higher levels of miR-21 in the poor prognosis group. There are some issues that could theoretically explain these diverging results. In the previous study [20], the patients were matched in respective groups with regard to age and sex, whereas in our present study we stratified the patients only with regard to outcomes. However, there were no differences in age and sex between the outcome groups in our study A technical difference is that we related the miRNA levels to both the spike-in and baseline values using the 2^-ΔΔCt^ formula, whereas Stammet *et al*. chose to relate only to the mean values of the spike-ins (2^-ΔCt^). When we analysed our data accordingly, however, the results were not altered. Another difference between our study and that of Stammet *et al*. is that we used only one spike-in control, whereas they used three. In our experience, the spike-in controls vary very little in relation to one another when added together, and we do not believe that this aspect of the studies can explain the diverging results.

Our study has some limitations. First, the long period between tissue collection and analysis of the samples might have affected the quality of the samples. However, miRNA is very stable, both at room temperature and after repetitive cycles of freeze-thawing. In our present study, all samples were thawed only once and stored at -80°C until the analysis [28]. Second, a general problem in miRNA research in plasma is the deficiency of well-validated endogenous controls to compensate for differences in RNA input. Regarding the cardiac arrest syndrome, the systemic inflammatory response and secondary effects of most organ systems makes it even more difficult to find a valid control for RNA load. Hence, in concordance with most of the published studies in this field, our data were not normalized to RNA input.

Before plasma miR-124 can be used for prognostication in the clinical setting, several technical aspects have to be handled. First, the preparation of samples and the PCR technique pose some limitations. RNA preparation, cDNA synthesis and PCR are time-consuming processes, and the validity of the results is highly dependent on meticulous handling of the samples. Second, in order to minimize imprecision, well-validated controls should be used. An endogenous control for RNA load in the setting of cardiac arrest is lacking, however, and more research should be focused on this area. Furthermore, it is important to observe whether the cutoff levels are totally dependent on both the endogenous control and the spike-in used in a particular study. Interestingly, several new non-PCR techniques for miRNA quantification in plasma, such as the nanopore device and methodology, are currently being developed and may overcome some of these issues [29].

## Conclusion

Our data suggest that the brain-enriched miRNA miR-124 is a promising, novel biomarker for predicting the prognosis for hypothermia-treated patients after cardiac arrest, with a calculated ROC AUCs of 0.87 at 24 hours and 0.89 at 48 hours. Our findings need to be validated prospectively in a larger cohort, which we plan to do as a substudy of the Target Temperature Management after Cardiac Arrest Trial [30].

## Key message

•‘The brain-enriched miRNA miR-124 is a promising, novel biomarker for the prediction of prognosis in hypothermia-treated patients after cardiac arrest.

## Abbreviations

ALT: Alanine aminotransferase; AUC: Area under the curve; CI: Confidence interval; CPC: Cerebral Performance Category; CRP: C-reactive protein; miRNA: microRNA; NSE: Neuron-specific enolase; qPCR: Quantitative polymerase chain reaction; ROC: Receiver operating characteristic; ROSC: Return of spontaneous circulation; TnT: Troponin T.

## Competing interests

The authors declare that they have no competing interests.

## Authors’ contributions

PG designed the study, analysed the data and drafted and revised most of the manuscript. OG processed the samples and drafted the manuscript with a particular focus on the Materials and methods section. MR recruited patients into the study, contributed to the data interpretation and critically revised the manuscript. TC made contributions to the design of the study, drafted the neurology-oriented sections and critically revised the manuscript. MA recruited patients into the study, contributed to the data interpretation and critically revised the manuscript. BO assisted in designing the study and critically revised the manuscript. HF contributed to the design of the study, drafted parts of the text and critically revised the manuscript. DE participated in the design of the study and critically revised the manuscript. All authors read and approved the final manuscript.

## Supplementary Material

Additional file 1 **Supplementary materials and methods data.** Laboratory work is described in more detail. Click here for file

Additional file 2: Figure S1 Screening of 20 plasma microRNAs at 48 hours after cardiac arrest. After Bonferroni correction, no significant differences were observed between the outcome groups for any of the microRNAs. The horizontal lines represent median values. Click here for file

Additional file 3: Figure S2 MicroRNA levels at admission relative to the spike-in Sp2. No differences between the outcome groups were observed. The horizontal lines represent median values. Click here for file
