# Supplementary material for: The brain-enriched microRNA miR-124 in plasma predicts neurological outcome after cardiac arrest
Source: Crit Care. 2014 Mar 3;18(2):R40. doi: 10.1186/cc13753 (PMC4057474; doi:10.1186/cc13753)
Supplement: Additional file 1 — Supplementary materials and methods data. Laboratory work is described in more detail. [file cc13753-S1.doc]

**Supplementary data to the materials and methods section.**

*RNA-isolation and cDNA-synthesis*

The blood samples were centrifuged immediately after collection followed by aspiration of the plasma supernatant, which was quickly frozen and stored at -80°C. The samples were thawed once, aliquoted and then refrozen until analysis. For total RNA-isolation, plasma was mixed with TRIzol LS (Invitrogen, Carlsbad, CA) in a 1:3 ratio and the samples were homogenized by vortexing >30 s. RNA was then isolated with the miRNeasy kit (Qiagen, Hilden, Germany) according to the manufacturer’s instructions. For normalization purposes, 1 μl of UniSP RNA Spike-in mix/sample (Exiqon, Vedbaek, Denmark) was added to the TRIzol before preparation of the samples. cDNA was synthesized with the miRCURY LNA Universal RT cDNA synthesis kit (Exiqon) using a fixed volume of RNA preparation in each reaction according to the manufacturer’s instructions. When preparing RNA from plasma, the yields are insufficient for proper quantitation with e.g. NanoDrop. Therefore, equal volumes of RNA preparation, rather than equal RNA amounts, was used as input in the cDNA synthesis

*qRT-PCR*

Custom qPCR-panels including 20 miRNAs were used for screening purposes according to the manufacturer’s instructions (Exiqon) on a StepOnePlus Real-Time PCR System (Applied Biosystems, Carlsbad, CA). A melt curve was performed after each PCR. An internal calibration control was used to compensate for run-to-run variations. Raw Ct-values were normalized against the mean of the spike-in controls Sp2 and Sp4 to adjust for differences in RNA extraction and reverse transcription efficiencies and expressed relative to the mean of the baseline samples using the formula 2^-ddCt^.

Since the qPCR panels did not include a negative control or duplicates, the results were confirmed by qRT-PCR in 10 µl duplicate reactions using Fast SYBR Green Master Mix (Applied Biosystems, Carlsbad, CA) and LNA primer sets (Exiqon) specific for the brain-enriched hsa-miR-124a, hsa-miR-21, hsa-miR-9, hsa-miR-128, the liver-specific hsa-miR-122, the cardiac-specific miR-208b and the inflammation-associated hsa-miR-146a according to the protocol of the manufacturer on a StepOnePlus Real-Time PCR System (Applied Biosystems, Carlsbad, CA). All PCR reactions yielded a single peak on the melt curve, indicating acceptable specificity of the primers. Negative controls were added on each plate and in case of contamination in these wells the plate was rerun. The threshold cycle (Ct) was defined where all assays were in the log linear phase and the threshold was above background for all assays. Samples where no amplification could be detected were defined as having a Ct of 35.
