# Supplementary figures and images for: The brain-enriched microRNA miR-124 in plasma predicts neurological outcome after cardiac arrest
Source: Crit Care. 2014 Mar 3;18(2):R40. doi: 10.1186/cc13753 (PMC4057474; doi:10.1186/cc13753)

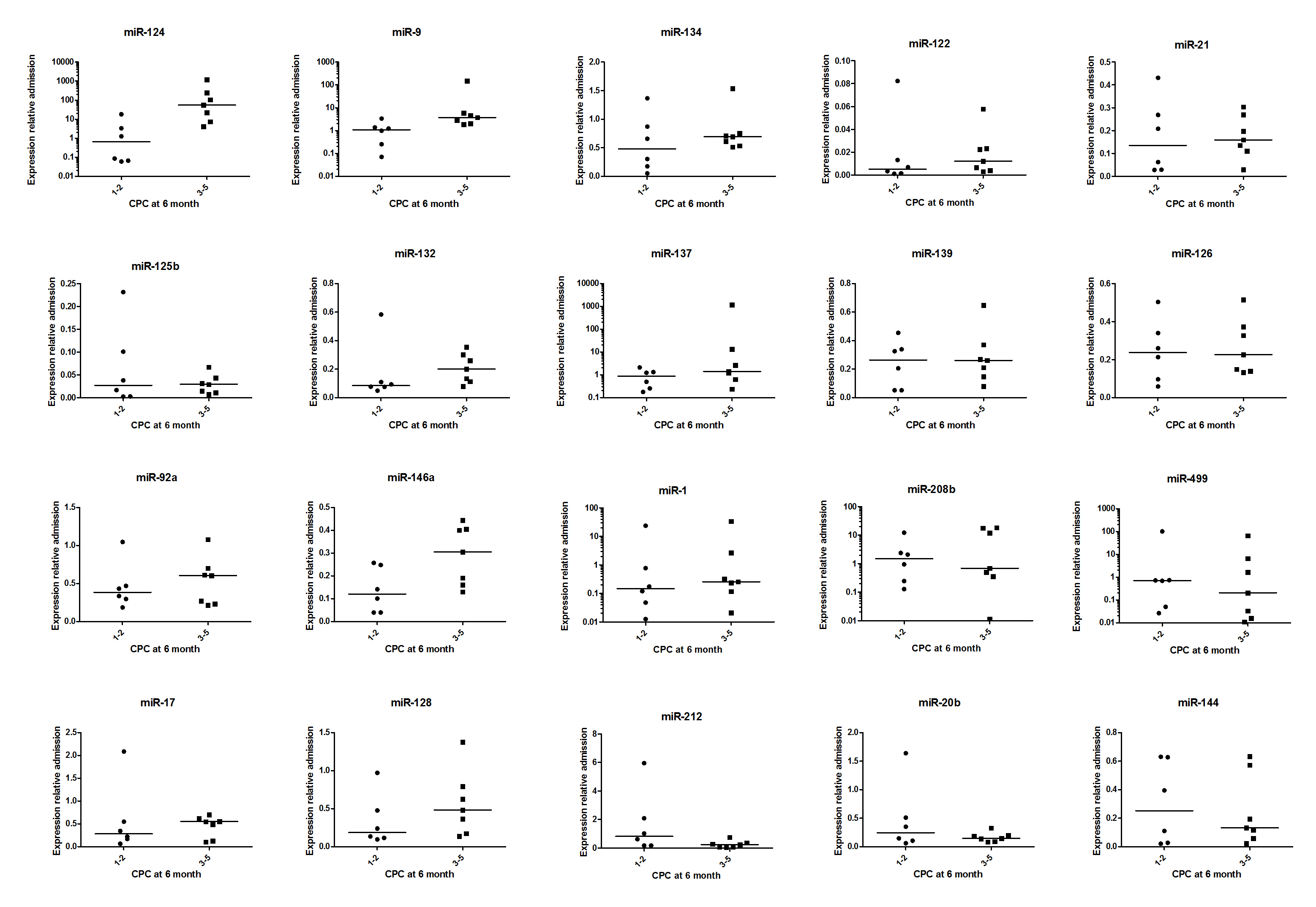

Supplement: Additional file 2: Figure S1 — Screening of 20 plasma microRNAs at 48 hours after cardiac arrest. After Bonferroni correction, no significant differences were observed between the outcome groups for any of the microRNAs. The horizontal lines represent median values. [file cc13753-S2.jpeg]

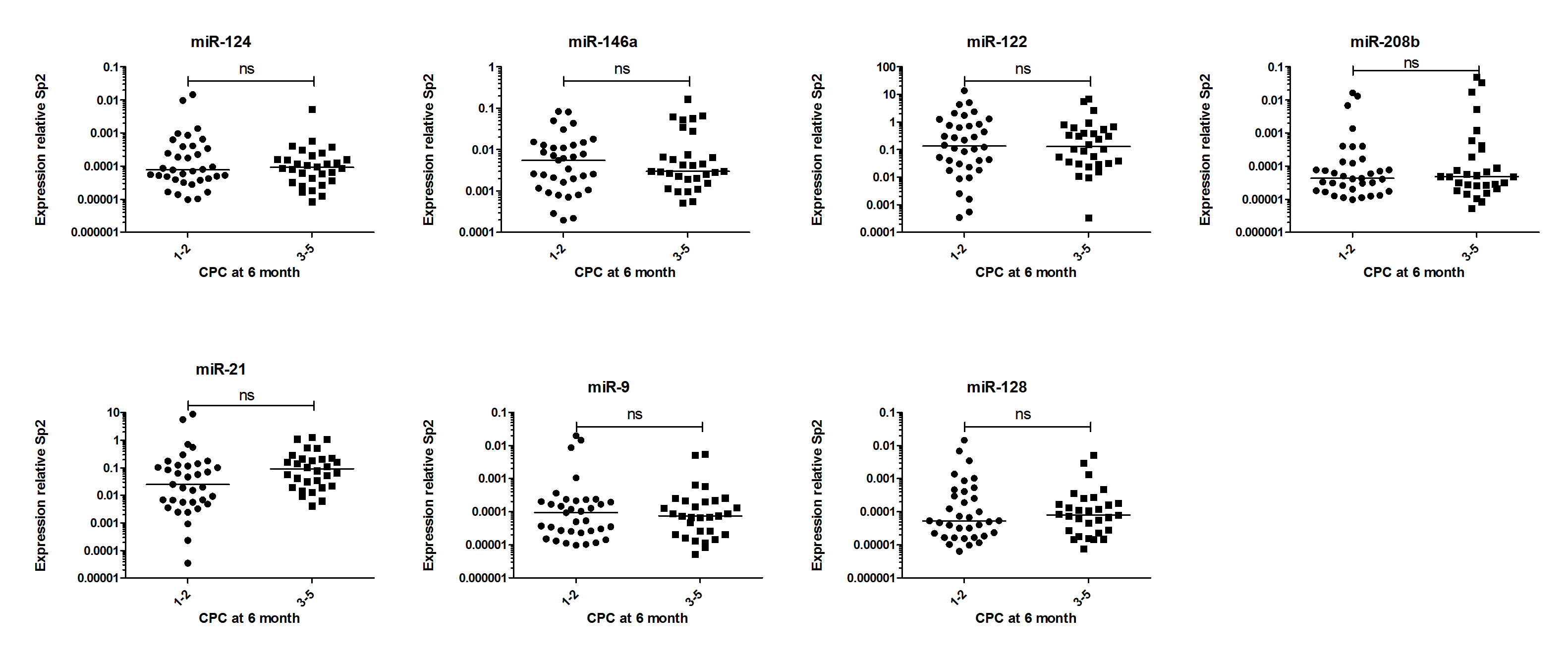

Supplement: Additional file 3: Figure S2 — MicroRNA levels at admission relative to the spike-in Sp2. No differences between the outcome groups were observed. The horizontal lines represent median values. [file cc13753-S3.jpeg]
